# Vaginal Hysterectomy at the Time of Total Colpocleisis: A Single-Center Experience

**DOI:** 10.7759/cureus.56574

**Published:** 2024-03-20

**Authors:** Riza Dur, Ayse Yalcinkaya Yilmaz, Betul Ahat, Mine Kanat Pektas

**Affiliations:** 1 Obstetrics and Gynecology, School of Medicine, Afyonkarahisar Health Sciences University, Afyonkarahisar, TUR

**Keywords:** postmenopausal women, quality of life, pelvic organ prolapse, hysterectomy, colpocleisis

## Abstract

Objective: This study aims to evaluate the five-year experience of a single center regarding the total colpocleisis procedure.

Methods: This is a retrospective review of 24 women who underwent total colpocleisis at the study center between January 2017 and January 2023. Every participant was informed about this study, and written consent was obtained from each participant who then took Pelvic Floor Distress Inventory-20 (PFDI-20), Body Appreciation Scale-2 (BAS-2) and Decision Regret Scale (DRS) questionnaires consecutively.

Results: Eight patients (33.3%) underwent total colpocleisis, whereas 16 patients (66.7%) had concomitant colpocleisis and vaginal hysterectomy. The number of total colpocleisis cases did not change significantly with respect to the past years (p=0.117). The patients who underwent total colpocleisis and the patients who had concurrent colpocleisis and hysterectomy were statistically similar with respect to age, gravidity, chronic disease, blood group, American Society of Anesthesiologists classification, anesthesia type, surgery timing and preoperative and postoperative hemoglobin values (p>0.05 for all). Operative time was significantly shorter in patients who had colpocleisis alone (p=0.001). Both patient groups were also statistically similar in aspects of blood loss, transfusion need, hospital stay, postoperative complications and follow-up time as well as PFDI-20, BAS-2 and DRS scores (p>0.05 for all). Endometrial atrophy (56.3%), endometrial hyperplasia (18.8%) and adenomyosis (12.5%) were the most common histopathological findings detected in vaginal hysterectomy specimens.

Conclusion: The combination of vaginal hysterectomy and total colpocleisis appears as a safe and efficient approach which does not contribute to the surgery-related morbidity despite the significantly longer operative time.

## Introduction

Pelvic organ prolapse can be defined as the downward herniation of pelvic organs including bladder, rectum, uterus and sometimes small intestine as well as the anterior and posterior vaginal wall [1]. This frequently encountered gynecological problem affects 40% of postmenopausal women, and it has been estimated that about 11% of the women would undergo surgery for prolapse or incontinence by 80 years of age [2,3].

Current management of pelvic organ prolapse consists of observation, pelvic floor muscle training, pessary use and surgery [4]. Surgical treatment of pelvic organ prolapse can be performed as a reconstructive or obliterative procedure. Reconstructive surgery is based on the maintenance of the pelvic anatomy and functions, whereas obliterative surgery depends on the closure of the vaginal canal [1,5].

Colpocleisis is the surgical obliteration of the vagina which emerges as a more substantial and less invasive procedure for pelvic organ prolapse [6,7]. This surgery can be regarded as an option for women who are no longer sexually active or cannot tolerate a longer and more invasive procedure [3,6]. Total colpocleisis depicts the suturation of anterior and posterior vaginal walls to each other after the complete removal of the vaginal epithelium [7,8]. On the other hand, partial colpocleisis, also named as LeFort, procedure refers to the formation of the longitudinal vaginal septum by approximating anterior and posterior vaginal walls which have been stripped of their epithelial tissue [9,10]. The original description of colpocleisis procedure does not refer to vaginal hysterectomy, but hysterectomy and colpocleisis can be performed concurrently [11,12]. Partial colpocleisis is preferred if the uterus is to be kept at its place. Either partial or total colpocleisis can be carried out in case of post-hysterectomy prolapse [7,11]. Yet, data about the concurrent implementation of vaginal hysterectomy and colpocleisis are limited and contradictory [6,8]. Therefore, this study has been designed to evaluate the five-year experience of a single center about total colpocleisis procedure.

## Materials and methods

The present study was approved by the Ethical Committee of Afyonkarahisar Health Sciences University Hospital where it was undertaken from January 1, 2023, to June 1, 2023 (grant no: 01.12.2023-2023/496). This is a retrospective review of 24 women who underwent total colpocleisis at the study center between January 2018 and January 2023. Total colpocleisis was performed after the patients refused the offer for using vaginal pessaries. Every participant was informed about this study, and written consent was obtained from each participant who then took Pelvic Floor Distress Inventory-20 (PFDI-20), Body Appreciation Scale-2 (BAS-2) and Decision Regret Scale (DRS) questionnaires consecutively. Data related to the patients' clinical and operative characteristics were obtained from medical records.

Total colpocleisis procedure

After the induction of anesthesia and administration of antibiotics, the patient was put in the dorsal lithotomy position. Candy-cane leggings were used to achieve better positioning and, thus, provide optimal visualization. Then, pelvic examination was made, and a sterile Foley catheter was applied to identify the bladder neck. A mixture of lidocaine and epinephrine was injected to ease dissection and ensure hemostasis. Afterward, sharp and/or blunt dissection was made, and vaginal epithelium was removed by a quadrant-based circumferential approach which required the preservation of vaginal epithelium extending beyond 1 cm proximity to the bladder and 3 cm proximity to the perineal body. The maintenance of vaginal epithelium extending beyond 1 cm proximity to the bladder allowed both voiding and the insertion of a mid-urethral sling when needed. On the other hand, sparing vaginal epithelium extending beyond 3 cm proximity to the perineal body permitted the reconstruction of the perineal body. Tissue repair was carried out by placing absorbable sutures in a purse-string manner. Several layers of purse-string repair were made to procure tissue imbrication, keep the potential space at minimum and, thus, overcome the prolapse completely. Before the transverse closure of the vaginal epithelium, cystoscopy was performed, and the patency of the urethra was demonstrated.

Vaginal hysterectomy

Vaginal hysterectomy was performed according to modified Heaney technique in 16 patients. This technique started with making an incision around the vaginal wall and cervix which would allow the bladder to be separated from the uterus. After the anterior and posterior peritoneum were opened, uterosacral and cardinal ligaments were cut and ligated, respectively. Uterine vasculature was ligated, and then uterine fundus was exposed so that tuboovarian and round ligaments were ligated. Ultimately, the uterus was removed. Afterward, the round ligament, uterosacral and cardinal peduncles were fixated to the vaginal mucosa; reperitonization was done; and vaginal mucosa was closed. Vaginal hysterectomy was planned preoperatively. Indications for vaginal hysterectomy included abnormal uterine bleeding (n=9), endometrial hyperplasia (n=3), adenomyosis (n=2), uterine leiomyoma (n=1) and cervical intraepithelial neoplasia (n=1).

Questionnaires

Developed by Barber et al. [13], Pelvic Floor Distress Inventory-20 (PFDI-20) consists of 20 items which are categorized under Pelvic Organ Prolapse Distress Inventory-6 (POPDI-6), Colorectal-Anal Distress Inventory-8 (CRADI-8) and Urinary Distress Inventory-6 (UDI-6) [13]. Each question begins with a "yes" or "no" response. If the answer is "yes," the severity of pelvic symptoms in the past three months has been designated on a four-point Likert scale with responses differing from 0 point (not at all) to 4 points (quite a bit). Each of the scores of POPDI-6, CRADI-8 and UDI-6 change between 0 and 100, and these scores were added to yield the total score which alters between 0 and 300. Higher scores reflect higher pelvic distress for PFDI-20 questionnaire which has been validated in the Turkish language [14].

Developed by Tylka and Wood-Barcalow, Body Appreciation Scale-2 (BAS-2) consists of 10 items which are based on a five-point Likert scale with responses ranging from 1 point (never) to 5 points (always) [15]. The total score of BAS-2 differs between 10 and 50, and higher scores indicate higher levels of body appreciation. This popular scale of one’s love and respect for his/her body has been adapted to the Turkish language [16].

Developed by Brehaut et al., Decision Regret Scale (DRS) includes five items which depend on a five-point Likert scale with responses altering between 1 point (strongly agree) to 5 points (strongly disagree) [17]. Zero points indicate no regrets, while 100 points indicate a high degree of regret. This scale has been validated in the Turkish language [18].

Statistical analysis

Collected data were analyzed by Statistical Package for Social Sciences version 21.0 (SPSS IBM, Armonk, NY, USA). Continuous variables were expressed as mean ± standard deviation or median, and categorical variables were denoted as number or percentage where appropriate. Student t-test, Mann-Whitney U test and chi-square test were used for the comparisons. Two-tailed p values <0.05 were accepted to be statistically significant.

## Results

**Figure 1:**
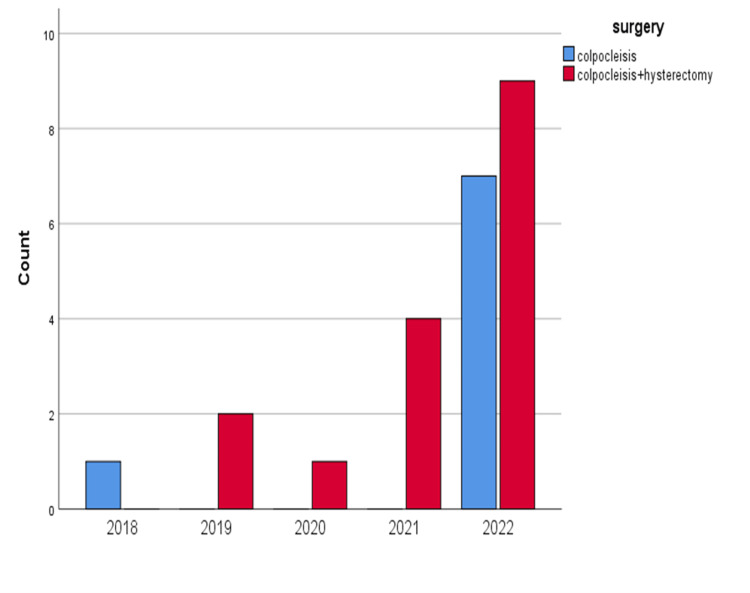
Number of total colpocleisis cases each year

Eight patients (33.3%) underwent total colpocleisis, whereas 16 patients (66.7%) had total colpocleisis and vaginal hysterectomy synchronously. Figure 1 shows the number of total colpocleisis cases each year. Accordingly, the number of total colpocleisis cases did not change significantly with respect to the past years (p=0.117).

Table 1 shows the sociodemographic and clinical characteristics of the patients. The patients who underwent total colpocleisis and the patients who had concurrent colpocleisis and hysterectomy were statistically similar in aspect of age, gravidity and chronic disease (p>0.05 for all).

**Table 1:** Clinical characteristics of the participants

	Total colpocleisis	Vaginal hysterectomy+ Total colpocleisis	p
	(n=8)	(n=16)	
Age (years)	69.13±6.03	72.56±8.14	0.303
Gravidity	3.63±0.74	3.81±0.91	0.620
Body mass index (kg/m^2^)	28.58±2.46	28.59±1.78	0.989
Chronic disease	6 (75.0%)	6 (75.0%)	0.698

Table 2 displays the operative characteristics of the participants. The patients who underwent total colpocleisis and the patients who had concurrent colpocleisis and hysterectomy were statistically similar with respect to the American Society of Anesthesiologists classification, anesthesia type, surgery timing and preoperative and postoperative hemoglobin values (p>0.05 for all). Operative time was significantly shorter in patients who had colpocleisis alone (p=0.001). Both patient groups were also statistically similar in aspects of blood loss, transfusion need, hospital stay and postoperative complications (p>0.05 for all).

**Table 2:** Operative characteristics of the participants *p<0.05 was accepted to be statistically significant.

	Total colpocleisis (n=8)	Vaginal hysterectomy+ Total colpocleisis (n=16)	p
American Society of Anesthesiologists			
Class I	0 (0.0%)	1 (6.2%)	0.449
Class II	3 (37.5%)	9 (56.3%)	0.387
Class III	5 (62.5%)	6 (37.5%)	0.216
General anesthesia	6 (75.0%)	13 (81.2%)	0.722
Regional anesthesia	2 (25.0%)	3 (18.8%)	0.725
Elective surgery	8 (100.0%)	13 (81.2%)	0.513
Urgent surgery	0 (0.0%)	3 (18.8%)	0.526
Operative time (min)	35.0±4.6	69.7±8.8	0.001*
Estimated blood loss	139.4±77.4	115.6±87.9	0.524
Preoperative hemoglobin (g/dl)	11.65±1.36	10.58±1.12	0.961
Postoperative hemoglobin (g/dl)	11.61±1.90	11.26±1.27	0.212
Need for transfusion	0 (0.0%)	1 (6.2%)	0.470
Duration of hospital stay (days)	2.75±0.71	3.44±1.79	0.310
Postoperative complications	1 (12.5%)	2 (12.4%)	0.723
Vaginal bleeding	1 (12.5%)	0 (0.0%)	0.148
Paralytic ileus	0 (0.0%)	1 (6.2%)	0.223
Intractable hypertension	0 (0.0%)	1 (6.2%)	0.223

Only one patient who underwent colpocleisis and vaginal hysterectomy synchronously (6.2%) required the transfusion of two erythrocyte suspensions. In addition, one patient who received total colpocleisis (12.5%) also had trans-obturator tape for the treatment of stress urinary incontinence. Endometrial atrophy (n=9, 56.3%), endometrial hyperplasia (n=3, 18.8%) and adenomyosis (n=2, 12.5%) were the most common histopathological findings detected in vaginal hysterectomy specimens (Table 3).

**Table 3:** Histopathological reports of the participants undergoing vaginal hysterectomy

	Number (%)
Endometrial atrophy	9 (56.3%)
Endometrial hyperplasia	3 (18.8%)
Adenomyosis	2 (12.5%)
Leiomyoma	1 (6.2%)
Cervical intraepithelial lesion	1 (6.2%)
Total	16 (100.0%)

Table 4 describes that the patients who underwent total colpocleisis and the patients who received concurrent colpocleisis and hysterectomy had statistically similar follow-up time as well as POPDI-6, CRADI-8, UDI-6, PFDI-20, BAS-2 and DRS scores (p>0.05 for all).

**Table 4:** Questionnaire results of the participants

	Total colpocleisis (n=8)	Vaginal hysterectomy+ Total colpocleisis (n=16)	p
Follow-up time (months)	12.38±1.26	17.75±1.27	0.321
Pelvic Organ Prolapse Distress Inventory-6	23.5±10.3	28.1±11.7	0.352
Colorectal-Anal Distress Inventory-8	28.5±12.2	30.8±10.1	0.635
Urinary Distress Inventory-6	33.3±11.7	34.5±11.1	0.800
Pelvic Floor Distress Inventory-20	85.3±29.7	93.4±24.8	0.486
Body Appreciation Scale-2	41.6±2.8	42.5±3.7	0.561
Decision Regret Scale	20.9±8.9	25.3±5.4	0.147

## Discussion

In this study, the patients who underwent total colpocleisis and the patients who had concurrent colpocleisis and hysterectomy were statistically similar with respect to anesthesia type, surgery timing and preoperative and postoperative hemoglobin values. Operative time was significantly shorter in patients who had colpocleisis alone. Both patient groups were also statistically similar in aspects of blood loss, transfusion need, hospital stay, postoperative complications and follow-up time as well as PFDI-20, BAS-2 and DRS scores.

Colpocleisis is an obliterative surgical procedure which provides an effective and substantial treatment for women who complain about pelvic organ prolapse [19]. Traditionally, this surgery has been considered a suitable option for elderly women who have comorbidities. The rationale behind this consideration is to attenuate the exposure to anesthetic agents, reduce the operation time and, thus, minimize surgery-related adverse outcomes [20,21]. Even though beneficial and hazardous outcomes of colpocleisis have been well defined, data about the concomitant implementation of vaginal hysterectomy and colpocleisis are limited. In fact, the main reason for performing hysterectomy at the time of colpocleisis is to avoid cancer and/or any possible delay in its diagnosis as endometrial cancer is the most common gynecological cancer. However, the addition of vaginal hysterectomy to colpocleisis would lengthen surgery time and, thus, might contribute to operative morbidities such as bleeding, need for transfusion and prolonged exposure to anesthesia as well as postoperative problems such as increased risk for infection and thromboembolism as well as latency in recovery time [22-25].

A retrospective analysis of 92 patients who underwent total colpocleisis with high levator plication reported that 37 patients (40.2%) also had vaginal hysterectomy simultaneously. Concurrent vaginal hysterectomy was found to be associated with a significant increase in absolute change of hematocrit and the need for transfusion. However, there was no significant difference between the patient groups in the aspect of surgical complications. The objective cure rate was 97.8% after a median follow-up of 12 months, while the subjective cure rate was 90.3% after a median follow-up of 24 months. Consequently, concomitant colpocleisis and hysterectomy were associated with higher blood loss and transfusion requirement [22].

Another retrospective review of 694 women who had surgery for pelvic floor reconstruction claimed that the rate of total or partial colpocleisis was 25.4%. The women who were exposed to concurrent colpocleisis and vaginal hysterectomy had a higher hemoglobin decrease and red blood cell loss than women undergoing colpocleisis alone. Although postoperative hemoglobin fall was the least in patients who had only colpocleisis, the addition of vaginal hysterectomy would be more likely to result in more intraoperative blood loss [23].

A similar retrospective review of 245 patients undergoing colpocleisis declared that operation time was significantly longer, and blood loss was significantly higher in case both colpocleisis and vaginal hysterectomy were performed synchronously. Similarly, the risk of venous thromboembolism was significantly higher in patients undergoing concurrent colpocleisis and hysterectomy. However, the increase in the risks of prolonged surgery, blood loss and thromboembolism were no longer significant after confounding factors such as age, body mass index and chronic diseases were controlled. The most common postoperative complication was urinary tract infection which affected 34.7% of the patients [24].

A multicenter study investigated 152 patients who had total or partial colpocleisis procedure. This investigation found the mean age of the patients as 79 years, and the objective cure rates were 82% and 73% at the third month and first-year control examinations, respectively. It was also specified that all pelvic symptom scores improved significantly at these controls, and 95% of the patients were either satisfied or very satisfied with the outcome of their surgery. The blood loss in the total colpocleisis group was significantly greater than in the partial colpocleisis group, and thus, the authors favored partial colpocleisis over total colpocleisis [25].

The retrospective analysis of a database included 1,027 women who underwent vaginal closure. About 87% of the patients had colpocleisis alone, while hysterectomy and colpocleisis were performed concomitantly in 13% of the patients. The surgery time was significantly longer, and postoperative complications were significantly more frequent in patients undergoing concurrent vaginal hysterectomy and colpocleisis. Hysterectomy was highlighted as an independent risk factor for complications related to vaginal obliteration. Urinary tract infection was the most common postoperative complication as it was observed in 4.3% of the patients [26].

As for the present study, 33.3% of the patients underwent total colpocleisis and 66.7% of the patients had total colpocleisis and vaginal hysterectomy synchronously. Although the surgery time was significantly shorter for women who had total colpocleisis alone, total colpocleisis only and concomitant colpocleisis and vaginal hysterectomy groups were statistically similar with respect to surgery time, blood loss and postoperative complications.

In this study, the women undergoing colpocleisis combined with vaginal hysterectomy are inclined to maintain their appreciation for their bodies and their satisfaction with the obliterative procedure. Moreover, symptoms related to pelvic floor dysfunction did not change significantly in women who had concomitant colpocleisis and vaginal hysterectomy. Interestingly, urinary tract infection did not occur as a postoperative complication in this cohort. This finding can be attributed to the meticulous administration of antibiotics.

As a result, it can be put forward that the combination of vaginal hysterectomy and total colpocleisis appears as a safe and efficient approach which does not contribute to surgery-related morbidity despite the significantly longer operative time. Hence, these findings should be interpreted carefully as their power is limited by the relatively small study cohort and retrospective study design. The heterogeneity in surgical skills and qualifications also might have caused bias. Moreover, the application of only six questionnaires might have limited the generalization of data related to postoperative well-being.

When there is a consideration for avoiding the risk of uterine cancer in the future, vaginal hysterectomy might be performed in women who have already been scheduled for colpocleisis. In fact, the risk of coinciding with a preinvasive or malignant lesion in the uterus alters between 0.8% and 2.9% during the repair of pelvic organ prolapse [27-29]. Based on this low risk, a decision analysis should be made to choose colpocleisis alone over concomitant hysterectomy in women older than 40 years [12]. Our findings suggest that concomitant vaginal hysterectomy can be performed safely and efficiently at the time of colpocleisis if there is a concern for a possible delay or negligence in the diagnosis of a uterine malignancy in the future. Further research has been warranted to determine the efficacy and safety of the concurrent implementation of total colpocleisis and vaginal hysterectomy and establish the indications for this surgical approach.

## Conclusions

In this study, the women undergoing colpocleisis combined with vaginal hysterectomy are inclined to maintain their appreciation for their bodies and their satisfaction with the obliterative procedure. Moreover, symptoms related to pelvic floor dysfunction did not change significantly in women who had concomitant colpocleisis and vaginal hysterectomy. Interestingly, urinary tract infection did not occur as a postoperative complication in this cohort. This finding can be attributed to the meticulous administration of antibiotics.

As a result, it can be put forward that the combination of vaginal hysterectomy and total colpocleisis appears as a safe and efficient approach which does not contribute to surgery-related morbidity despite the significantly longer operative time. Hence, these findings should be interpreted carefully as their power is limited by a relatively small study cohort, retrospective study design and variations in surgical techniques and surveying preferences.
